# Identification and characterization of conserved lncRNAs in human and rat brain

**DOI:** 10.1186/s12859-017-1890-7

**Published:** 2017-12-28

**Authors:** Dan Li, Mary Qu Yang

**Affiliations:** MidSouth Bioinformatics Center and Joint Bioinformatics Ph.D. Program, University of Arkansas at Little Rock and University of Arkansas for Medical Sciences, 2801 S. University Avenue, Little Rock, AR 72204 USA

**Keywords:** Orthologous analysis, Long non-coding RNAs, Conserved lncRNAs, Animal model

## Abstract

**Background:**

Long noncoding RNAs (lncRNAs) are involved in diverse biological processes and play an essential role in various human diseases. The number of lncRNAs identified has increased rapidly in recent years owing to RNA sequencing (RNA-Seq) technology. However, presently, most lncRNAs are not well characterized, and their regulatory mechanisms remain elusive. Many lncRNAs show poor evolutionary conservation. Thus, the lncRNAs that are conserved across species can provide insight into their critical functional roles.

**Results:**

Here, we performed an orthologous analysis of lncRNAs in human and rat brain tissues. Over two billion RNA-Seq reads generated from 80 human and 66 rat brain tissue samples were analyzed. Our analysis revealed a total of 351 conserved human lncRNAs corresponding to 646 rat lncRNAs.

Among these human lncRNAs, 140 were newly identified by our study, and 246 were present in known lncRNA databases; however, the majority of the lncRNAs that have been identified are not yet functionally annotated. We constructed co-expression networks based on the expression profiles of conserved human lncRNAs and protein-coding genes, and produced 79 co-expression modules. Gene ontology (GO) analysis of the co-expression modules suggested that the conserved lncRNAs were involved in various functions such as brain development (*P*-value = 1.12E-2), nervous system development (P-value = 1.26E-3), and cerebral cortex development (P-value = 1.31E-2). We further predicted the interactions between lncRNAs and protein-coding genes to better understand the regulatory mechanisms of lncRNAs. Moreover, we investigated the expression patterns of the conserved lncRNAs at different time points during rat brain growth. We found that the expression levels of three out of four such lncRNA genes continuously increased from week 2 to week 104, which is consistent with our functional annotation.

**Conclusion:**

Our orthologous analysis of lncRNAs in human and rat brain tissues revealed a set of conserved lncRNAs. Further expression analysis provided the functional annotation of these lncRNAs in humans and rats. Our results offer new targets for developing better experimental designs to investigate regulatory molecular mechanisms of lncRNAs and the roles lncRNAs play in brain development. Additionally, our method could be generalized to study and characterize lncRNAs conserved in other species and tissue types.

**Electronic supplementary material:**

The online version of this article (10.1186/s12859-017-1890-7) contains supplementary material, which is available to authorized users.

## Background

Long non-coding RNAs (lncRNAs) act as regulators in diverse biological processes and are involved in many human diseases, including cancer. The expression alterations of some lncRNAs are associated with cancer patient survival [1]. The number of identified lncRNAs has been accumulating rapidly in recent years [2]. Despite the many efforts that have been made to predict how they function [3], presently, only a small fraction of lncRNAs are well characterized [4].

Evolutionarily conserved lncRNAs show stable and critical functions across species, despite their low number [5]. Chodroff et al. discovered four highly conserved lncRNAs in the mouse brain. The expression pattern of these lncRNAs further indicated their putative functions in vertebrate brain development [6]. Rats are one of the most widely used animal model organisms for elucidating drug mechanisms and studying chemical toxicity. Importantly, the genome and transcriptomic BodyMap of the rat have been generated recently [7]. Detailed investigation of the lncRNAs conserved between humans and rats can more accurately indicate the functions of lncRNAs and further guide the experimental studies of lncRNAs in rats.

Here, we develop a computational framework for the identification and annotation of conserved lncRNAs based on gene co-expression networks, lncRNA-protein interactions, and temporal expression patterns. More than 2 billion human and rat brain RNA sequencing reads from the Sequencing Quality Control (SEQC) consortium were processed. The lncRNAs identified by our integrative pipeline and annotated by Ensembl were combined to discover lncRNAs conserved between humans and rats. Further gene ontology (GO) analysis and lncRNA-protein interactions [8] of the enriched co-expressed gene modules indicated the potential functions of the lncRNAs. Our study represents a new method for investigating lncRNAs and provides insight into their regulation. The results can be used to design and guide experiments that aim to validate lncRNA functions in rats. This method can be applied to study conserved lncRNAs across other species and tissue types.

## Results

### Conserved lncRNAs in human and rat

**Fig. 1:**
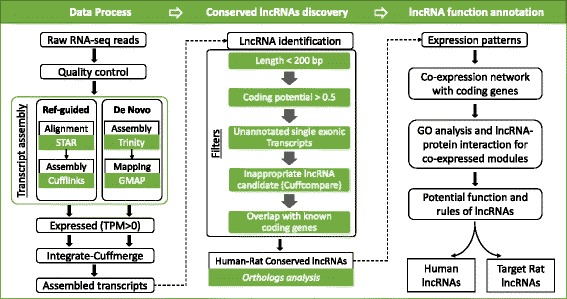
The workflow of conserved lncRNA identification and annotation

We developed a computational framework to systematically identify pair-wise conserved lncRNAs between humans and rats (Fig. 1, Methods). Over 2 billion RNA-Seq reads generated from 80 human and 66 rat brain tissue samples [7, 9] were processed and assembled utilizing our method. A coding-potential assessment of the assembled transcripts using lncScore [10] yielded 33,203 human and 53,782 rat lncRNA candidates. To reduce false positives that could be generated by assembly [11] and coding-potential [10, 12] methods, we applied several critical filters (Methods) to determine a high-confident lncRNA set. Finally, we attained 8150 human and 11,688 rat lncRNAs for conservation analysis. Of the human lncRNAs, 30.8% (2510/8150) overlapped with Ensembl lncRNA, and 95.6% (7791/8150) overlapped with lncRNAs in MiTranscriptome [2]. MiTranscriptome is a human lncRNA database derived from the computational analysis of RNA-Seq data from various cancer and tissue types and currently does not contain lncRNA annotations from other species. Thus, we combined our assembled lncRNAs and annotated lncRNAs from humans (13,258, version GRCh38.87) and rats (3267, version Rnor_6.0.87) using Ensembl for further conservation and function analysis. On the basis of orthologous analysis (Methods), we identified a total of 351 conserved human lncRNAs, consisting of 105 newly identified and 246 annotated sequences in Ensembl as well as 646 rat lncRNAs (574 new and 72 annotated).

**Fig. 2:**
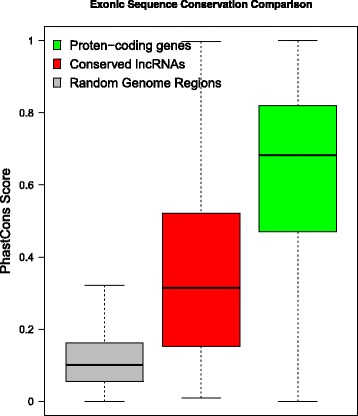
The sequence conservation of different types of human coding regions based on PhastCons scores

**Fig. 3:**
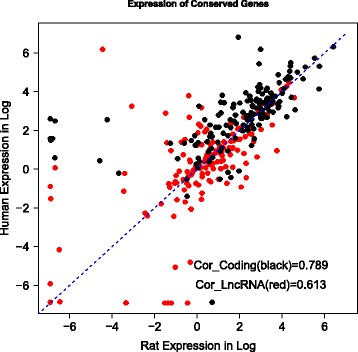
The comparison of the expression correlation of conserved lncRNA and protein-coding genes

Human and murine lineages diverged from each other approximately 90 million years ago. A previous study suggested that lncRNAs with different evolutionary ages show various sequence constraint patterns [5]. We assessed the sequence conservation between human and rat transcripts based on the PhastCons score [13]. As expected, the lncRNA conservation score was lower than that of the protein-coding genes but higher than that of the random sequence (Fig. 2). Notably, the score distributions of these lncRNAs conserved between humans and rats is consistent with the score distributions of the lncRNAs with an evolutionary age of 90 million years, as defined in a previous large-scale study. We also evaluated correlations of the expression of transcripts conserved between humans and rats. We found that the Spearman’s correlation coefficient was 0.61 and 0.79 for conserved lncRNAs and protein-coding genes, respectively (Fig. 3). A previous study showed that the correlations of conserved lncRNA and protein-coding gene expression between humans and a species with a divergence from humans of 90-million-years were approximately 0.4 and 0.8, respectively [5]. The higher lncRNA correlation (0.61 versus 0.4) we observed may be attributed to the incompleteness of lncRNA annotation in rat tissues, especially in tissue types other than brain.

### Co-expression network of conserved lncRNAs and protein-coding genes

**Fig. 4:**
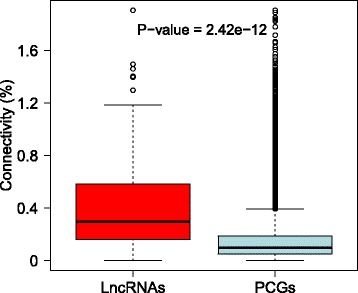
The connectivity of lncRNAs and protein-coding genes in the co-expression modules

Next, we measured the co-expression of the lncRNAs and the protein-coding genes, which can suggest their functional relatedness and potential regulatory relationship. Applying the weighted correlation network analysis (WGCNA) [14], we built a co-expression network on the basis of the expression levels of 351 conserved lncRNAs and 80,008 protein-coding transcripts in human brain tissue. Here, the protein-coding transcripts were obtained from the Ensembl database. As a result, 79 significant co-expression modules were revealed. With the exception of one very large module containing 9019 genes, the size of these modules ranged from 229 to 1509. Additionally, 238 conserved human lncRNAs were identified in the 70 co-expression modules. The connections between individual nodes, which represented either protein-coding genes or lncRNAs, were determined by expression correlation and topological overlap [14]. Furthermore, we computed the connectivity of each node, given by the degree of a node divided by the total degrees in an individual module. We found that conserved lncRNAs tended to have significantly higher connectivity than most of the protein-coding genes (Fig. 4, Wilcoxon test *P* = 2.43E-12), suggesting their potential to have central regulatory roles.

The coordinating expression of lncRNAs and protein-coding genes indicated their functional relevance. We performed a gene ontology (GO) analysis on the protein-coding genes of each module to discover their enriched GO terms and to infer the potential functions of the lncRNAs in the same module. We found that 56 of 79 co-expression modules were significantly associated with at least one biological process term (*P* < 0.05). Additionally, we calculated the interaction scores of the lncRNA and protein-coding gene pairs in the module using lncPro [8], a software tool used to predict interactions between lncRNAs and protein-coding genes. Based on the collective evidence from the co-expression analysis and interaction evaluations, we can infer the putative function of these lncRNAs.

**Fig. 5:**
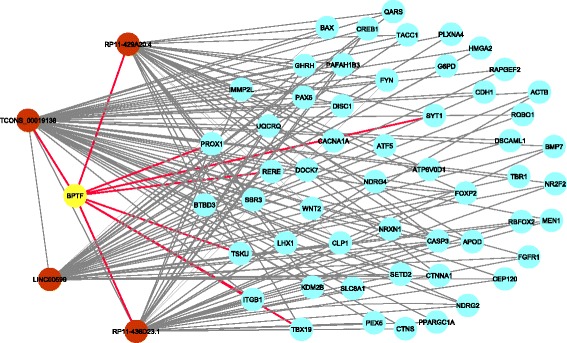
The subnetwork of brain development-related genes. Only the connections (edges) between lncRNAs and *BPTF* and other nodes in the subnetwork are displayed

**Fig. 6:**
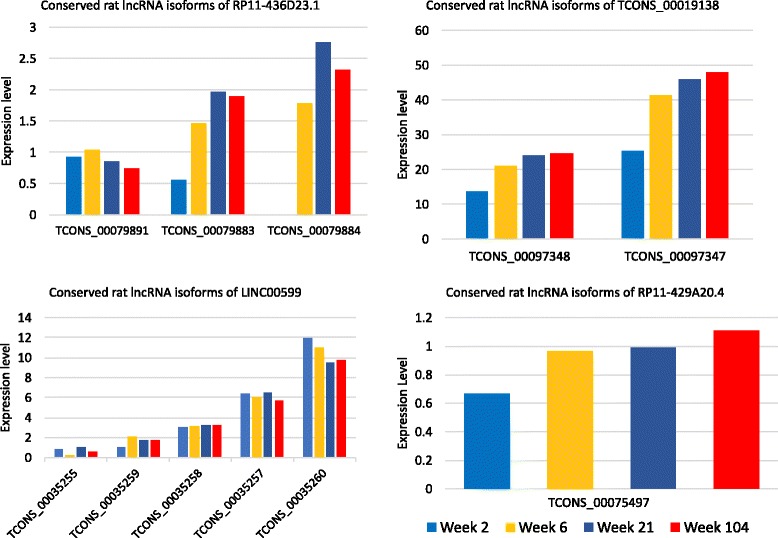
The expression patterns of conserved rat lncRNA isoforms in orthologous regions mapped from 4 human lncRNAs. The 4 human lncRNA genes were identified by co-expression analysis and by protein and lncRNA interaction prediction

As an example, one of the co-expression modules comprised 897 protein-coding transcripts and four lncRNAs. Three of the four lncRNAs, RP11-436D23.1, RP11-429A20.4, and LINC00599, were included in the Ensembl database, but their functions are uncharacterized. The fourth, TCONS_00019138, was newly identified by our study. The GO analysis on this module revealed a gene cluster consisting of 56 protein-coding genes that was significantly associated with brain development (*P* = 0.0112). The lncRNAs were connected to most of these 56 coding genes within the network, indicating their roles in brain development (Fig. 5). Moreover, we computed the interaction scores of the lncRNAs with the protein-coding genes in this gene cluster. The resulting scores suggest that all four lncRNAs likely interacted with *BPTF,* a protein-coding gene associated with Alzheimer disease and subplate neurons in the developing human brain (Fig. 5). Additionally, we assessed changes in the expression of the rat lncRNAs corresponding to the four human lncRNAs at different developmental stages: week 2, week 6, week 21, and week 104. Each conserved lncRNA family contained one or more isoforms (Fig. 6). Despite the fact that the expression of the isoforms in each family varied, we found that the expression levels of at least one isoform in each rat lncRNA family tended to continuously increase from week 2 to week 104 (Fig. 6). Importantly, the expression levels of these rat lncRNAs were significantly elevated from week 2 to week 6. (Fig. 6), which is a critical period for rat brain growth. A previous report suggested that by day 35, the rat brain reaches 95% of the adult brain weight and achieves maximum gray matter volume and cortical thickness [15]. Thus, we conclude that the four human lncRNAs function in brain development and that their conserved genes in rats, four newly identified rat lncRNAs, have conserved functional roles in brain development.

### Bidirectional lncRNA and protein-coding gene pairs

Bidirectional lncRNA protein-coding gene (PCG) pairs share the same promoter regions, which can indicate a functional relationship. Many bidirectional promoters that are associated with lncRNAs and PCGs were indicated to be associated with neuronal functions. Of the 233 human lncRNAs in the family, 41 lncRNAs were divergently transcribed from their adjacent protein coding genes, which were located at 2000 or fewer base pairs away. Furthermore, 16 of these 41 lncRNAs had the same neighboring protein-coding genes in rats. A subsequent GO analysis of 16 common protein-coding genes revealed 11 significant biological process terms. Notably, 10 of the 11 enriched biological process terms were associated with brain or neural functions in both humans and rats. Interestingly, none of the bidirectional lncRNA and protein-coding genes presented simultaneously in the co-expression modules that we identified in the previous steps, suggesting that lncRNAs exert a variety of regulatory mechanisms.

### Temporal expression of lncRNAs in rat brain over the lifespan

The lifespan of rats is approximately 2.6 years. The RNA-Seq data used in this study were generated from rat brain tissues at week 2, week 6, week 21, and week 104. A temporal expression analysis of 646 conserved rat lncRNAs showed that the expression levels of 48 lncRNAs consistently increased, whereas that of 57 decreased over the average rat’s lifespan. Moreover, we found that 63 conserved human lncRNA isoforms corresponded to 48 continuously up-regulated rat lncRNAs, and 126 human lncRNA isoforms corresponded to 57 continuously down-regulated rat lncRNAs. Most of these lncRNAs do not yet have a functional annotation. When searching lncRNAdb [16], a database that offers functional annotations of eukaryotic lncRNAs, we found the functional annotations of eight lncRNAs (Additional file 1: Table S1). Five of these lncRNAs have functions related to the brain [6, 17–21], and two lncRNAs [22–24] have roles in tumor development.

**Fig. 7:**
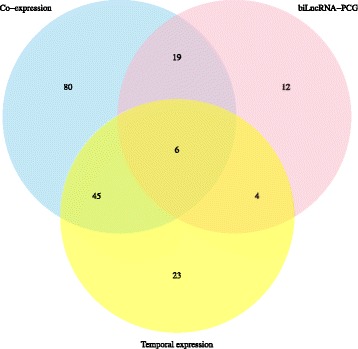
The functional annotations based on different methods. We used three methods to infer the putative function of conserved lncRNAs. Co-expression (blue circle) refers to the lncRNA functions that were suggested according to the co-expression modules and lncRNA-protein interaction prediction. biLncRNA-PCG (pink) refers to the lncRNAs that are divergently transcribed with their adjacent protein-coding genes. Temporal expression (yellow) refers the lncRNAs that have conserved rat lncRNA partners displaying consistently up−/down-regulated expression during rat development

In this study, we applied a co-expression network analysis and an lncRNA-protein interaction prediction to infer the putative functions of the conserved lncRNAs. We also investigated the temporal expression of lncRNAs in the rat brain and putative *cis*-regulation of bidirectional lncRNAs-PCG to complete and improve functional annotations. As a result, 81.1% (189/233) of 233 conserved lncRNA families were potentially annotated (Fig. 7, Additional file 1, List of conserved lncRNAs). Here, isoforms located in the same genomic region are considered to be an lncRNA family.

## Discussion

In this study, we used the lncRNAs identified by our method and those annotated by Ensembl to detect lncRNAs conserved between humans and rats. Based on the RNA-Seq data from human and rat brain tissues, we found that many Ensembl lncRNAs were not expressed in brain due to tissue-specific expression patterns of lncRNAs. Only 40% of the annotated conserved human lncRNAs were expressed with median transcripts per million (TPM > 0) in the brain tissue samples, compared to 79% expressed newly identified lncRNAs (Additional file 2: Figure S1). These results suggest that we identified more brain-specific lncRNAs. The conserved lncRNAs between humans and rats can benefit and further guide future studies.

The genomes of most eukaryotes are complex*.* One gene often contains multiple isoforms with varying structures resulting from alternative splicing. These complexities challenge the computational approaches for assembling the full-length transcripts [15]. The assemblers, such as Cufflinks and Trinity, tended to generate new isoforms belonging to the same gene family [2]. Rat gene annotation, especially that of lncRNAs, is largely incomplete. At present, only 3267 lncRNAs are annotated in Ensembl. Multiple lncRNAs may be located within the same conserved genomic region. For instance, RP11-472I20.3–001 is a human lncRNA located in chromosome 11. We found 3 annotated lncRNAs (red) and 5 assembled lncRNAs (black) located in the corresponding orthologous rat genome region. (Additional file 3: Figure S2). This finding explained why we obtained 351 conserved human lncRNAs corresponding to 646 rat lncRNAs in our study.

Despite various assembly methods that have been developed, detecting full-length transcripts from RNA-Seq data remains a challenge. The best-performing assembly method can only detect approximately 21% of full-length human protein-coding genes from RNA-Seq data in humans [11]. These partially detected transcripts can produce false positive lncRNA identification due to their incomplete coding sequence. Our integrative method enables the identification of more full-length lncRNAs. Additionally, the lncScore that we employed in our analysis showed higher accuracy than other methods, including CPAT, CNCI and PLEK for protein-coding potential assessments. To ensure the reliability of the downstream analysis, we applied stringent filters to further reduce false positives; however, this may have filtered out some true lncRNAs. Nevertheless, this improved assembly method will lead to more comprehensive and accurate lncRNA identification.

The method we proposed here focused on the characterization of conserved lncRNAs. Though the number of conserved lncRNAs represents only a small fraction of all lncRNAs, several studies have reported their functional importance. Thus, functional annotation of these lncRNAs could provide a critical understanding of conserved lncRNAs, which comprise an essential group of lncRNAs.

## Conclusions

In this study, we identified lncRNAs conserved in human and rat brain. We found that these conserved lncRNAs have important functional roles and tend to be more active than most protein-coding genes. The gene co-expression network analysis suggested the potential functions of the lncRNAs. Moreover, identification of the protein-coding genes that are highly likely to interact with lncRNAs yielded novel insights into the regulatory mechanisms of lncRNAs. Our results provide targets to investigate lncRNA functions and regulatory mechanisms using the rat model.

## Methods

### Transcript assembly

We processed and assembled raw RNA-Seq data utilizing an integrative method (Fig. 1 left panel). Our integrative method combined reference-guided and de novo assembly strategies, enabling a more comprehensive assembly of transcripts from RNA-Seq data. After QA/QC (quality assessment and quality control) using FASTQC (v0.10.1) and Trimmomatic (v0.36) [25], the low quality reads were removed. The remaining reads were assembled separately by STAR (v2.4.0)-Cufflinks (v2.2.1) and Trinity (v2.1.1)-GMAP (version 2015–12-31). Then, Cuffmerge was applied to integrate the expressed transcripts (TPM > 0) from STAR-Cufflinks and Trinity-GMAP.

### LncRNA identification

A series of stringent filters was adopted to distinguish lncRNAs from all assembled transcripts (Fig. 1 top middle panel). (i) LncScore (v1.0.2) [10] was used to remove transcripts of less than 200 bp and those having high (> 0.5) coding potential values. (ii) Cuffcompare was utilized to compare the assembled transcripts with existing gene annotations. The assembled transcripts were cataloged into specific types. We removed the transcripts that overlapped with an opposite DNA strand of known gene annotation, single-exonic transcripts without annotation, and transcripts that overlapped with protein-coding genes.

### Orthologous analysis

We utilized liftOver to compare the genome coordinates of human lncRNAs (hg38) to the rat genome (rn6) according to *hg38ToRn6.over.chain* (Fig. 1 bottom middle panel). Default parameters of liftOver were adopted. The rat lncRNAs located within or overlapping with conserved human genome regions were considered to be conserved pair-wise with human lncRNAs.

### Signed weighted co-expression network construction

The expression of the protein-coding transcripts and lncRNAs in all human samples was measured by TPM (kallisto, v0.43.0) [26]. The expression matrix was entered into the WGCNA (v1.51) to build the co-expression network. Accounting for both up- and down-regulation, we built a signed network with a minimum module size of 30 nodes (genes). After the gene module detection, the cutoff of the topological overlap of two nodes was set to 0.2 for further analysis, including degree assessment.

## Additional files

Additional file 1: List of conserved lncRNAs and **Table S1. **(DOCX 33 kb)

Additional file 2: Figure S1. The expression of conserved lncRNAs compared with the expression of non-conserved lncRNAs and protein-coding genes in human brain. (PDF 53 kb)

Additional file 3: Figure S2. Multiple rat lncRNAs locate in the orthologous region of a human lncRNA RP11-472I20.3–001. (PDF 120 kb)
