# Correction to: Identification and characterization of conserved lncRNAs in human and rat brain

**DOI:** 10.1186/s12859-018-2202-6

**Published:** 2018-05-24

**Authors:** Dan Li, Mary Qu Yang

**Affiliations:** MidSouth Bioinformatics Center and Joint Bioinformatics Ph.D. Program, University of Arkansas at Little Rock and University of Arkansas for Medical Sciences, 2801 S. University Avenue, Little Rock, AR 72204 USA

## Correction

After publication of the original article [1], it was noticed that the Acknowledgement statement was incorrect. The original statement reads:

“This project is supported by NIHR15GM114739, FDABAA-15-00121, AEDC grant #77138 and NIGMS P20GM103429.”

However, the correct Acknowledgement statement should instead read:

“This project is partially supported by NIHR15GM114739, AEDC grant #77138, NIGMS P20GM103429 and the Food and Drug Administration (FDA), contract No. HHSF223201510172C and HHSF223201610111C. However, the information contained herein represents the position of the author(s) and not necessarily that of the NIH and FDA.”
