# Supplementary material for: Identification and characterization of conserved lncRNAs in human and rat brain
Source: BMC Bioinformatics. 2017 Dec 28;18(Suppl 14):489. doi: 10.1186/s12859-017-1890-7 (PMC5751786; doi:10.1186/s12859-017-1890-7)
Supplement: Supplementary file 1 — List of conserved lncRNAs and Table S1. (DOCX 33 kb) [file 12859_2017_1890_MOESM1_ESM.docx]

The new conserved human lncRNAs.

| Chr | Start | End | Strand | Chr | Start | End | Strand | Chr | Start | End | Strand |
| --- | --- | --- | --- | --- | --- | --- | --- | --- | --- | --- | --- |
| chr1 | 31790458 | 31792769 | + | chr4 | 577060 | 580348 | + | chr13 | 50043587 | 50070643 | - |
| chr1 | 58785142 | 58901133 | + | chr4 | 577060 | 580348 | + | chr13 | 50043587 | 50081988 | - |
| chr1 | 1.9E+08 | 1.9E+08 | + | chr5 | 17403896 | 17442315 | + | chr13 | 93830153 | 94027894 | - |
| chr1 | 97927208 | 97979565 | - | chr5 | 72095893 | 72099067 | - | chr13 | 96085066 | 96089376 | - |
| chr1 | 97927208 | 98045379 | - | chr5 | 88654894 | 88660269 | - | chr14 | 21520820 | 21525648 | + |
| chr1 | 1.59E+08 | 1.59E+08 | - | chr5 | 1.41E+08 | 1.41E+08 | - | chr14 | 62117301 | 62134345 | + |
| chr1 | 1.59E+08 | 1.59E+08 | - | chr5 | 1.41E+08 | 1.41E+08 | - | chr14 | 62117383 | 62134345 | + |
| chr1 | 1.78E+08 | 1.78E+08 | - | chr5 | 1.41E+08 | 1.41E+08 | - | chr14 | 51966605 | 51969928 | - |
| chr1 | 1.78E+08 | 1.78E+08 | - | chr5 | 1.41E+08 | 1.41E+08 | - | chr14 | 62100393 | 62112789 | - |
| chr1 | 1.78E+08 | 1.78E+08 | - | chr5 | 1.42E+08 | 1.42E+08 | - | chr14 | 90398201 | 90408275 | - |
| chr2 | 5982813 | 5991168 | + | chr5 | 1.5E+08 | 1.5E+08 | - | chr14 | 1.01E+08 | 1.01E+08 | - |
| chr2 | 47906777 | 47908266 | + | chr6 | 7280928 | 7289931 | + | chr15 | 37099079 | 37136758 | + |
| chr2 | 47906827 | 47908266 | + | chr6 | 79234006 | 79236796 | + | chr15 | 37099395 | 37131985 | + |
| chr2 | 62415364 | 62501698 | + | chr6 | 1.66E+08 | 1.66E+08 | - | chr15 | 82750564 | 82757208 | + |
| chr2 | 70392692 | 70450854 | + | chr7 | 28952567 | 28959049 | + | chr15 | 82750566 | 82757208 | + |
| chr2 | 1.04E+08 | 1.05E+08 | + | chr7 | 41693947 | 41713182 | + | chr15 | 1.02E+08 | 1.02E+08 | + |
| chr2 | 1.78E+08 | 1.78E+08 | + | chr7 | 1.24E+08 | 1.24E+08 | + | chr16 | 49284331 | 49290678 | + |
| chr2 | 1.55E+08 | 1.55E+08 | - | chr7 | 30156879 | 30280964 | - | chr16 | 49284331 | 49290678 | + |
| chr2 | 1.55E+08 | 1.55E+08 | - | chr7 | 93962818 | 93968343 | - | chr16 | 66720837 | 66723611 | + |
| chr2 | 1.55E+08 | 1.55E+08 | - | chr7 | 96955131 | 97004278 | - | chr16 | 56189655 | 56191042 | - |
| chr2 | 1.55E+08 | 1.55E+08 | - | chr7 | 96955131 | 97012040 | - | chr16 | 82858359 | 83032216 | - |
| chr2 | 1.69E+08 | 1.69E+08 | - | chr7 | 1.05E+08 | 1.05E+08 | - | chr17 | 49369712 | 49370364 | + |
| chr2 | 1.82E+08 | 1.82E+08 | - | chr7 | 1.31E+08 | 1.31E+08 | - | chr17 | 79806230 | 79835452 | + |
| chr3 | 28576692 | 28758808 | + | chr9 | 1.24E+08 | 1.24E+08 | - | chr17 | 31531619 | 31538224 | - |
| chr3 | 28577098 | 28758808 | + | chr9 | 1.24E+08 | 1.24E+08 | - | chr17 | 36926096 | 36936655 | - |
| chr3 | 50261317 | 50262970 | + | chr10 | 73497478 | 73498968 | + | chr17 | 36926096 | 36936655 | - |
| chr3 | 1.28E+08 | 1.28E+08 | + | chr10 | 79382272 | 79389567 | + | chr17 | 45146159 | 45152252 | - |
| chr3 | 4979410 | 4985330 | - | chr11 | 95230831 | 95231154 | + | chr17 | 50052165 | 50055842 | - |
| chr3 | 42529660 | 42531493 | - | chr11 | 95231318 | 95234397 | + | chr19 | 29398657 | 29401259 | - |
| chr3 | 42531837 | 42537642 | - | chr11 | 1.07E+08 | 1.07E+08 | + | chr19 | 29488068 | 29497147 | - |
| chr3 | 42644079 | 42654313 | - | chr12 | 89585810 | 89601365 | + | chr21 | 33065808 | 33072428 | - |
| chr3 | 1.14E+08 | 1.14E+08 | - | chr12 | 71785643 | 71822106 | - | chr21 | 33551704 | 33555120 | - |
| chr3 | 1.18E+08 | 1.18E+08 | - | chr12 | 72262540 | 72265165 | - | chrX | 24540236 | 24550686 | + |
| chr3 | 1.52E+08 | 1.52E+08 | - | chr12 | 72271610 | 72272551 | - | chrX | 24542201 | 24550686 | + |
| chr3 | 1.61E+08 | 1.61E+08 | - | chr13 | 19199295 | 19208612 | - | chrY | 2978764 | 2982562 | - |

The annotated conserved human lncRNAs.

| **Gene Name** | **Transcript Name** | **Gene Name** | **Transcript Name** | **Gene Name** | **Transcript Name** |
| --- | --- | --- | --- | --- | --- |
| LINC01006 | LINC01006-001 | LINC00237 | LINC00237-003 | LINC01089 | LINC01089-003 |
| MIRLET7BHG | MIRLET7BHG-001 | RP11-418J17.3 | RP11-418J17.3-001 | RP11-123O10.4 | RP11-123O10.4-001 |
| RP11-374M1.5 | RP11-374M1.5-001 | LINC00511 | LINC00511-009 | RP11-123O10.3 | RP11-123O10.3-001 |
| MEG3 | MEG3-005 | RP11-768G7.2 | RP11-768G7.2-003 | LINC01089 | LINC01089-008 |
| MEG3 | MEG3-007 | LINC01140 | LINC01140-004 | MIR9-3HG | MIR9-3HG-001 |
| LINC01551 | LINC01551-003 | RP4-621F18.2 | RP4-621F18.2-002 | LINC01089 | LINC01089-012 |
| H19 | H19-002 | LINC01140 | LINC01140-007 | CTD-2555A7.2 | CTD-2555A7.2-001 |
| AC005082.12 | AC005082.12-001 | RP11-779P15.2 | RP11-779P15.2-001 | RP11-123O10.3 | RP11-123O10.3-002 |
| LINC01412 | LINC01412-001 | LINC01140 | LINC01140-005 | RP11-429A20.4 | RP11-429A20.4-001 |
| MIR137HG | MIR137HG-004 | RP11-14D22.2 | RP11-14D22.2-001 | LINC01089 | LINC01089-004 |
| H19 | H19-001 | DUBR | DUBR-005 | RP11-444D3.1 | RP11-444D3.1-004 |
| H19 | H19-005 | LINC00632 | LINC00632-001 | RP11-444D3.1 | RP11-444D3.1-001 |
| AC007879.2 | AC007879.2-002 | RP11-736K20.6 | RP11-736K20.6-002 | RMST | RMST-001 |
| RP11-436D23.1 | RP11-436D23.1-005 | NEAT1 | NEAT1-002 | RP13-941N14.1 | RP13-941N14.1-001 |
| RP11-161I10.1 | RP11-161I10.1-001 | NEAT1 | NEAT1-001 | LINC01089 | LINC01089-011 |
| LINC00607 | LINC00607-006 | RP11-867G23.3 | RP11-867G23.3-001 | LINC01089 | LINC01089-006 |
| FTX | FTX-003 | RP11-18H21.3 | RP11-18H21.3-001 | LINC01089 | LINC01089-009 |
| AC007383.3 | AC007383.3-001 | RP11-73G16.2 | RP11-73G16.2-001 | LINC01089 | LINC01089-010 |
| LINC00237 | LINC00237-002 | MALAT1 | MALAT1-002 | LINC01089 | LINC01089-007 |
| RP11-145M4.1 | RP11-145M4.1-001 | RP11-663P9.1 | RP11-663P9.1-001 | RMST | RMST-002 |
| MEG3 | MEG3-002 | LINC00461 | LINC00461-016 | SNHG23 | SNHG23-012 |
| LINC01351 | LINC01351-001 | LINC00461 | LINC00461-015 | SNHG23 | SNHG23-015 |
| LINC01006 | LINC01006-007 | SNHG18 | SNHG18-002 | LINC01578 | LINC01578-003 |
| GAS5 | GAS5-020 | RP11-319E12.1 | RP11-319E12.1-001 | MEG9 | MEG9-004 |
| LINC00472 | LINC00472-004 | CARMN | CARMN-002 | SNHG23 | SNHG23-013 |
| MEG3 | MEG3-001 | LINC01438 | LINC01438-001 | MEG3 | MEG3-026 |
| PTPRD-AS1 | PTPRD-AS1-001 | RP11-371M22.1 | RP11-371M22.1-001 | LINC01578 | LINC01578-005 |
| LINC01105 | LINC01105-003 | LINC00461 | LINC00461-008 | SNHG23 | SNHG23-014 |
| LINC01105 | LINC01105-001 | LINC00461 | LINC00461-006 | LINC01578 | LINC01578-006 |
| LINC00390 | LINC00390-001 | CTD-3224K15.2 | CTD-3224K15.2-001 | MEG3 | MEG3-027 |
| GAS5 | GAS5-016 | RP11-247C2.2 | RP11-247C2.2-001 | MEG3 | MEG3-029 |
| AC007383.3 | AC007383.3-002 | LINC00461 | LINC00461-004 | LINC01578 | LINC01578-008 |
| MIR99AHG | MIR99AHG-008 | LINC00599 | LINC00599-002 | LINC01578 | LINC01578-001 |
| AC099754.1 | AC099754.1-001 | PVT1 | PVT1-007 | CRNDE | CRNDE-009 |
| AC005013.5 | AC005013.5-001 | PVT1 | PVT1-009 | MIR9-3HG | MIR9-3HG-008 |
| AC104655.3 | AC104655.3-001 | LINC00599 | LINC00599-003 | RP11-27M9.1 | RP11-27M9.1-001 |
| LINC01351 | LINC01351-002 | LINC00599 | LINC00599-001 | CRNDE | CRNDE-001 |
| MIR34A | MIR34A-002 | MIR124-2HG | MIR124-2HG-005 | MIR9-3HG | MIR9-3HG-006 |
| RP11-456H18.2 | RP11-456H18.2-001 | LINC01605 | LINC01605-004 | MIR9-3HG | MIR9-3HG-012 |
| RP11-89K21.1 | RP11-89K21.1-001 | MIR124-2HG | MIR124-2HG-001 | MIR9-3HG | MIR9-3HG-005 |
| H19 | H19-003 | MEG3 | MEG3-013 | MIR9-3HG | MIR9-3HG-007 |
| AC007879.3 | AC007879.3-001 | MIR124-2HG | MIR124-2HG-004 | AC140912.1 | AC140912.1-001 |
| AC144525.1 | AC144525.1-001 | MEG3 | MEG3-020 | RP11-247C2.2 | RP11-247C2.2-002 |
| RP11-343J18.2 | RP11-343J18.2-001 | MEG3 | MEG3-019 | RP4-561L24.3 | RP4-561L24.3-001 |
| MIR99AHG | MIR99AHG-003 | PVT1 | PVT1-003 | CTD-2050B12.1 | CTD-2050B12.1-001 |
| H19 | H19-015 | MEG3 | MEG3-014 | CTD-2012K14.6 | CTD-2012K14.6-001 |
| MIRLET7BHG | MIRLET7BHG-007 | KB-173C10.2 | KB-173C10.2-002 | RP11-491F9.6 | RP11-491F9.6-001 |
| LINC01117 | LINC01117-001 | MIR124-2HG | MIR124-2HG-002 | RP11-701H24.4 | RP11-701H24.4-001 |
| MIR217HG | MIR217HG-001 | MEG3 | MEG3-021 | RP11-386M24.6 | RP11-386M24.6-002 |
| H19 | H19-006 | PVT1 | PVT1-001 | LINC00922 | LINC00922-002 |
| LINC00578 | LINC00578-004 | MIR124-2HG | MIR124-2HG-003 | RP4-555D20.2 | RP4-555D20.2-001 |
| RP11-444D3.1 | RP11-444D3.1-006 | RP11-472I20.3 | RP11-472I20.3-001 | RP11-679B19.2 | RP11-679B19.2-001 |
| H19 | H19-014 | MIR100HG | MIR100HG-009 | RP11-268G13.1 | RP11-268G13.1-001 |
| AC023137.2 | AC023137.2-001 | MIR100HG | MIR100HG-001 | RP11-491F9.8 | RP11-491F9.8-001 |
| RP11-160N1.10 | RP11-160N1.10-001 | RAB30-AS1 | RAB30-AS1-007 | MIR22HG | MIR22HG-012 |
| RP11-2L8.1 | RP11-2L8.1-001 | MIR100HG | MIR100HG-008 | MIR193BHG | MIR193BHG-001 |
| RP11-107I14.5 | RP11-107I14.5-001 | MIR100HG | MIR100HG-006 | MIR22HG | MIR22HG-011 |
| MEG3 | MEG3-006 | CTD-2589M5.5 | CTD-2589M5.5-001 | MIR193BHG | MIR193BHG-003 |
| AC104655.3 | AC104655.3-002 | RP11-736K20.6 | RP11-736K20.6-001 | MIR22HG | MIR22HG-002 |
| U73166.2 | U73166.2-003 | MIR670HG | MIR670HG-001 | RP11-436D23.1 | RP11-436D23.1-010 |
| MEG3 | MEG3-004 | MIR100HG | MIR100HG-004 | RP11-204E9.1 | RP11-204E9.1-002 |
| FTX | FTX-004 | URB1-AS1 | URB1-AS1-001 | RP11-472I20.3 | RP11-472I20.3-004 |
| MIR22HG | MIR22HG-006 | MIR4453 | MIR4453-001 | MALAT1 | MALAT1-012 |
| AC140912.1 | AC140912.1-002 | CTD-2012K14.6 | CTD-2012K14.6-002 | MALAT1 | MALAT1-013 |
| CTD-2008L17.1 | CTD-2008L17.1-001 | CASC15 | CASC15-006 | MALAT1 | MALAT1-009 |
| LINC01140 | LINC01140-008 | FGF14-AS2 | FGF14-AS2-001 | LINC01140 | LINC01140-201 |
| LINC00237 | LINC00237-004 | LINC00632 | LINC00632-005 | MALAT1 | MALAT1-011 |
| RP4-806M20.3 | RP4-806M20.3-001 | RP11-28H5.2 | RP11-28H5.2-001 | MALAT1 | MALAT1-005 |
| NEAT1 | NEAT1-005 | RP11-222G7.2 | RP11-222G7.2-001 | CTD-2105E13.16 | CTD-2105E13.16-001 |
| CARMN | CARMN-008 | MIR22HG | MIR22HG-018 | AC005753.1 | AC005753.1-001 |
| MIR99AHG | MIR99AHG-019 | RP11-212P7.2 | RP11-212P7.2-001 | TEX41 | TEX41-073 |
| MIR222HG | MIR222HG-002 | MIR219A2 | MIR219A2-001 | CH507-513H4.4 | CH507-513H4.4-001 |
| MIR222HG | MIR222HG-001 | MIR22HG | MIR22HG-016 | KCNIP4-IT1 | KCNIP4-IT1-001 |
| LINC00632 | LINC00632-004 | MIR22HG | MIR22HG-019 | LINC00602 | LINC00602-001 |
| RP11-193M21.1 | RP11-193M21.1-001 | RP11-126K1.9 | RP11-126K1.9-001 | MIR325HG | MIR325HG-009 |
| FTX | FTX-006 | RP11-352M15.2 | RP11-352M15.2-001 | CH507-513H4.6 | CH507-513H4.6-001 |
| MIR137HG | MIR137HG-002 | MALAT1 | MALAT1-008 | RP3-510D11.4 | RP3-510D11.4-001 |
| MIR99AHG | MIR99AHG-016 | NEAT1 | NEAT1-003 | RP4-665J23.1 | RP4-665J23.1-009 |
| MIR137HG | MIR137HG-003 | MALAT1 | MALAT1-010 | CASC15 | CASC15-008 |
| RP11-706J10.2 | RP11-706J10.2-001 | MALAT1 | MALAT1-014 | LINC00598 | LINC00598-017 |
| LINC01109 | LINC01109-001 | CARMN | CARMN-012 | RP4-665J23.1 | RP4-665J23.1-007 |
| RP11-1055B8.9 | RP11-1055B8.9-001 | AC007950.2 | AC007950.2-001 | RP4-665J23.1 | RP4-665J23.1-008 |

Table S1. The functional annotations of eight conserved human lncRNAs with consistent expression patterns in mouse.

| LncRNA_ID | NAME | Species besides human | Expression pattern | Function description |
| --- | --- | --- | --- | --- |
| ENSG00000223403 | MEG9 | Rat/Mouse/Sheep | UP | Some Meg9 miRNAs have a known function, including one known to be important in brain development [s1]. |
| ENSG00000280650 | KCNIP4-IT1 | Pig | UP | Identified as a RNA that is more highly expressed in adult brain than fetal brain; Expression in human and pig was similar. Mostly restricted to adult brain, highest expression in cerebellum. |
| ENSG00000249859 | PVT1 | NA | UP | Expression levels positively correlated with Myc protein expression in human primary tumors [s2]. Generates antiapoptotic activity in CRC [s3]. |
| ENSG00000230590 | FTX | Mouse | UP | Mutations in Ftx leads to changes of expression in the X-inactivation center and decreases Xist levels. |
| ENSG00000214548 | MEG3 | Mouse/Sheep | DOWN | Expressed in the nucleus accumbens of normal human brains and upregulated in this brain region in heroin abusers [s4]. Meg3 knock-out increased expression of VEGF (Vascular Endothelial Growth Factor) signaling pathway genes in the brain, suggesting the RNA has a role in the control of vascularization, and therefore may function as a tumor suppressor in part by inhibiting angiogenesis [s5]. |
| ENSG00000245532 | NEAT1 | Rat/Mouse/Dog/Cattle | DOWN | Expressed in the nucleus accumbens of normal human brains and upregulated in this brain region in heroin abusers[s4]. Transcription of Neat1 is essential for the assembly, maintenance and structural integrity of paraspeckles [s6]. |
| ENSG00000255794 | RMST | Mouse | UP & DOWN | Expressed in specific regions of embryonic and post-natal mouse brain [s7]. Expression in embryonic brain includes in “ventral midbrain where dopaminergic neurons are formed. So Rmst may be a marker for developing dopaminergic neurons; 2kb transcript also expressed in adult brain [s8]. |
| ENSG00000234741 | GAS5 | Rat/Mouse/Chicken | UP & DOWN | Necessary and sufficient for growth arrest in human peripheral blood T-cells. Functions by controlling apoptosis and the cell cycle in lymphocytes [s9]. |

[s1] Schratt GM, Tuebing F, et al. A brain-specific microRNA regulates dendritic spine development, Nature 2006.

[s2] Tseng YY, Moriarity BS, Gong W, et al. PVT1 dependence in cancer with MYC copy-number increase, Nature 2014

[s3] Takahashi Y, Sawada G, Kurashige J, et al. Amplification of PVT-1 is involved in poor prognosis via apoptosis inhibition in colorectal cancers, British Journal of Cancer 2014.

[s4] Michelhaugh, S. K., Lipovich, L., Blythe, J., Jia, H., Kapatos, G., & Bannon, M. J. (2011). Mining Affymetrix microarray data for long noncoding RNAs: altered expression in the nucleus accumbens of heroin abusers. Journal of Neurochemistry, 116(3), 459–466.

[s5] Gordon FE, Nutt CL, Cheunsuchon P, et al. Increased Expression of Angiogenic Genes in the Brains of Mouse Meg3-Null Embryos. Endocrinology. 2010;151(6):2443-2452. doi:10.1210/en.2009-1151.

[s6] Clemson CM, Hutchinson JN, Sara SA, et al. An Architectural Role for a Nuclear Non-coding RNA: NEAT1 RNA is Essential for the Structure of Paraspeckles. Molecular cell. 2009;33(6):717-726. doi:10.1016/j.molcel.2009.01.026.

[s7] Chodroff RA, Goodstadt L, Sirey TM, et al. Long noncoding RNA genes: conservation of sequence and brain expression among diverse amniotes. Genome Biology. 2010;11(7):R72. doi:10.1186/gb-2010-11-7-r72.

[s8] Uhde CW, Vives J, Jaeger I, Li M. Rmst Is a Novel Marker for the Mouse Ventral Mesencephalic Floor Plate and the Anterior Dorsal Midline Cells. Riley B, ed. PLoS ONE. 2010;5(1):e8641. doi:10.1371/journal.pone.0008641.

[s9] Mirna Mourtada-Maarabouni, Vanessa L. Hedge, Lucy Kirkham, et al. Growth arrest in human T-cells is controlled by the non-coding RNA growth-arrest-specific transcript 5 (GAS5), Journal of Cell Science, 2008
