# Supplementary figures and images for: Identification and characterization of conserved lncRNAs in human and rat brain
Source: BMC Bioinformatics. 2017 Dec 28;18(Suppl 14):489. doi: 10.1186/s12859-017-1890-7 (PMC5751786; doi:10.1186/s12859-017-1890-7)

Expression of Conserved Human lncRNAs

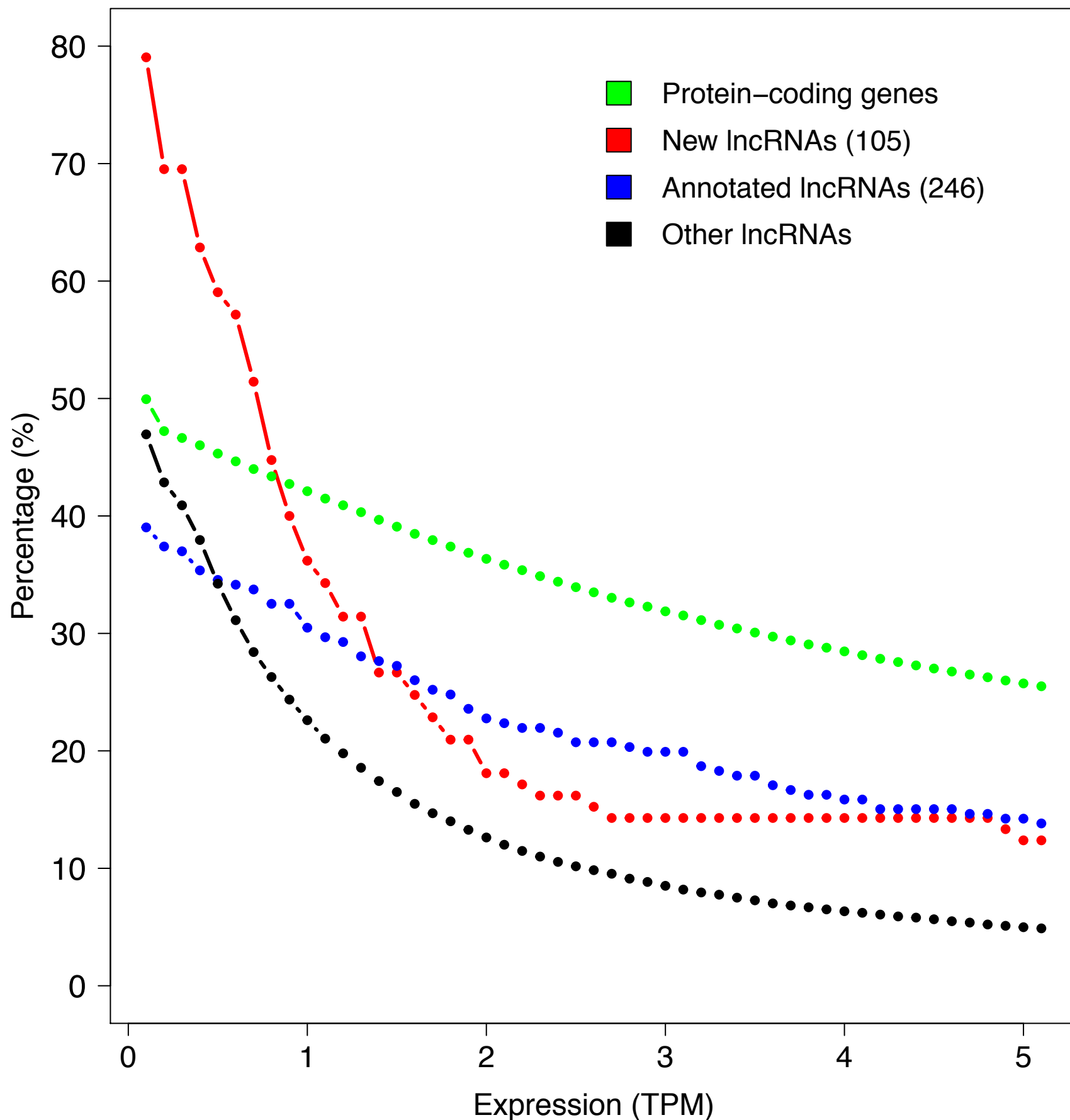

Supplement: Supplementary file 2 — The expression of conserved lncRNAs compared with the expression of non-conserved lncRNAs and protein-coding genes in human brain. (PDF 53 kb) [file 12859_2017_1890_MOESM2_ESM.pdf]
