# Supplementary material for: Identification and characterization of conserved lncRNAs in human and rat brain
Source: BMC Bioinformatics. 2017 Dec 28;18(Suppl 14):489. doi: 10.1186/s12859-017-1890-7 (PMC5751786; doi:10.1186/s12859-017-1890-7)
Supplement: Supplementary file 3 — Multiple rat lncRNAs locate in the orthologous region of a human lncRNA RP11-472I20.3–001. (PDF 120 kb) [file 12859_2017_1890_MOESM3_ESM.pdf]

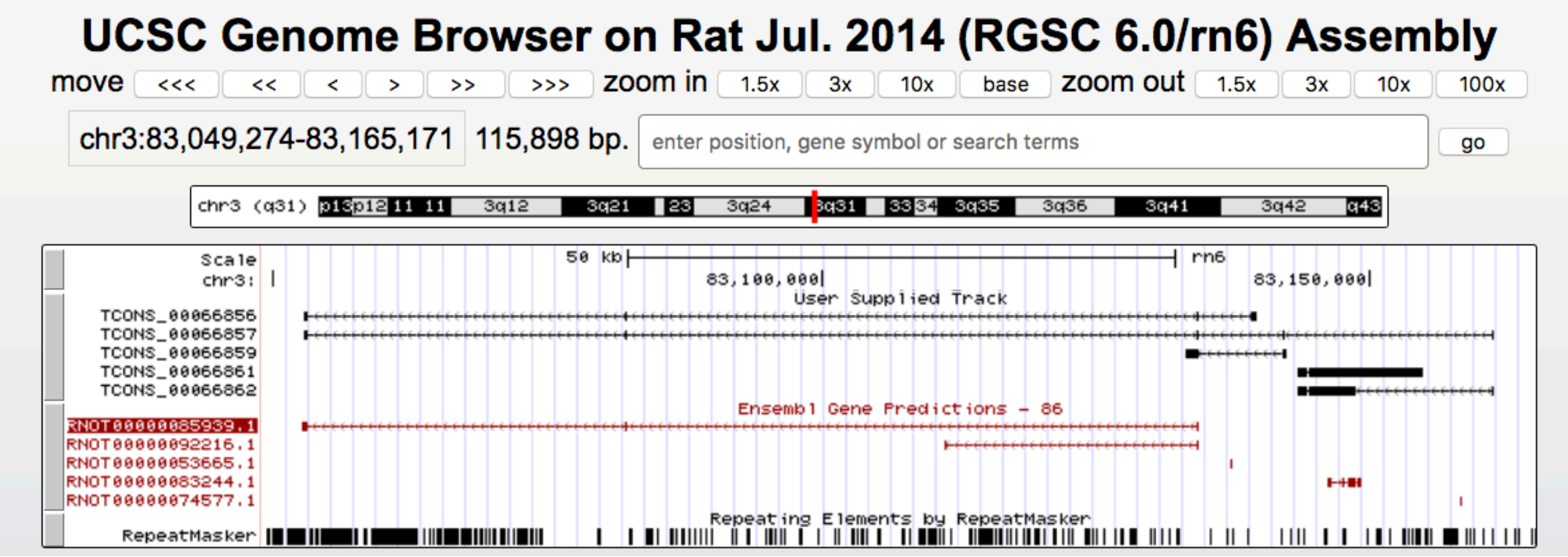
UCSC Genome Browser on Rat Jul. 2014 (RGSC 6.0/rn6) Assembly
move <<< << < > >> >>> zoom in 1.5x 3x 10x base zoom out 1.5x 3x 10x 100x
chr3:83,049,274-83,165,171 115,898 bp.
enter position, gene symbol or search terms
go
chr3 (q31)
3q12 3q21 23 3q24 3q31 33 34 3q35 3q36 3q41 3q42 q43
Scale
50 kb
rn6
chr3:
83,100,000
83,150,000
User Supplied Track
TCONS_00066856
TCONS_00066857
TCONS_00066859
TCONS_00066861
TCONS_00066862
Ensembl Gene Predictions - 86
RNOT00000085939.1
RNOT00000092216.1
RNOT00000053665.1
RNOT00000083244.1
RNOT00000074577.1
Repeating Elements by RepeatMasker
RepeatMasker
